# Artificial intelligence-assisted photodynamic diagnosis and photodynamic therapy against cancer

**DOI:** 10.3389/fonc.2026.1932816

**Published:** 2026-08-25

**Authors:** Jinju Huang, Siu Kan Law, Albert Wing Nang Leung, Chuanshan Xu

**Affiliations:** 1 Intensive Care Unit Ward 1, The Affiliated Panyu Central Hospital, Guangzhou Medical University, Guangzhou, China; 2 Independent Researcher, Hong Kong, China; 3 School of Graduate Studies, Lingnan University, Hong Kong, China; 4 Guangzhou Municipal and Guangdong Provincial Key Laboratory of Molecular Target & Clinical Pharmacology, The NMPA and State Key Laboratory of Respiratory Disease, School of Pharmaceutical Sciences, Guangzhou Medical University, Guangzhou, China

**Keywords:** artificial intelligence, cancer diagnosis, cancer therapy, photodynamic diagnosis, photodynamic therapy, photosensitizer

## Abstract

Artificial intelligence (AI) comprises advanced computational systems designed to simulate selected aspects of human cognitive functions, such as learning, reasoning, and perception. While AI does not fully replicate human cognition, its analytical processing of large datasets through “machine learning (ML)” and “deep learning (DL)” has increasingly been applied in medicine, including photodynamic diagnosis (PDD) and photodynamic therapy (PDT). In oncology, these modalities are increasingly used for early cancer detection, tumor margin delineation, and personalized therapeutic planning. These applications involve AI-PDD/PDT workflows, dosimetry, predictive modeling, and translational opportunities across Western and Chinese medicine. The working process of AI-PDD/PDT systems encompasses treatment workflows, dosimetry optimization, real-time monitoring, and outcome prediction. AI-driven systems standardize photosensitizer preparation, reduce human error, and enhance reproducibility in PDT. Findings are synthesized conceptually across diverse studies, focusing on thematic integration of AI-assisted PDD/PDT mechanisms rather than quantitative pooling or statistical comparison of outcomes. Cancer applications include bladder, gastric, colorectal, and breast tumors, where AI-PDD/PDT improves diagnostic precision and enhances therapeutic efficacy. The rapid development of AI in recent years has extended into PDT, supporting fundamental research, clinical translation, and therapeutic innovation. AI-PDD/PDT demonstrates potential to enhance accuracy, safety, and efficiency through workflow optimization and predictive modeling. However, current evidence remains preliminary, and further systematic validation and clinical trials are required to substantiate these proposed benefits. Future milestones include refining the interface between AI and PDD/PDT and conducting prospective, multicenter trials to establish clinical utility.

## 1 Introduction

Artificial intelligence (AI) has become increasingly integrated into medical practice, enabling real-time analysis of large datasets and supporting clinical decision-making (1, 2). Machine learning (ML) and deep learning (DL) provide algorithmic frameworks and neural architectures to enhance diagnostic precision, risk stratification, and therapeutic optimization in oncology (3–5). These technologies have already demonstrated promise in tumor detection, segmentation, and classification across imaging modalities such as magnetic resonance imaging (MRI), computed tomography (CT), and fluorescence imaging (6, 7).

Photodynamic diagnosis (PDD) and photodynamic therapy (PDT) have advanced but face persistent limitations. Tumor visualization and margin delineation have been improved in PDD, but recurrence and progression risks remain significant in bladder, gastric, and peritoneal cancers (8, 9). PDT offers targeted eradication of malignant cells and has indicated efficacy in breast, colorectal, and gastric tumors (10–12). However, conventional PDT relies on empirical dosimetry, fixed wavelength activation, and retrospective outcome analysis, which restrict reproducibility and personalization. PDD and PDT struggle with challenges in clinical oncology, including variability in photosensitizer (PS) uptake, lack of standardized protocols, and insufficient multicenter validation.

**Figure 1:**
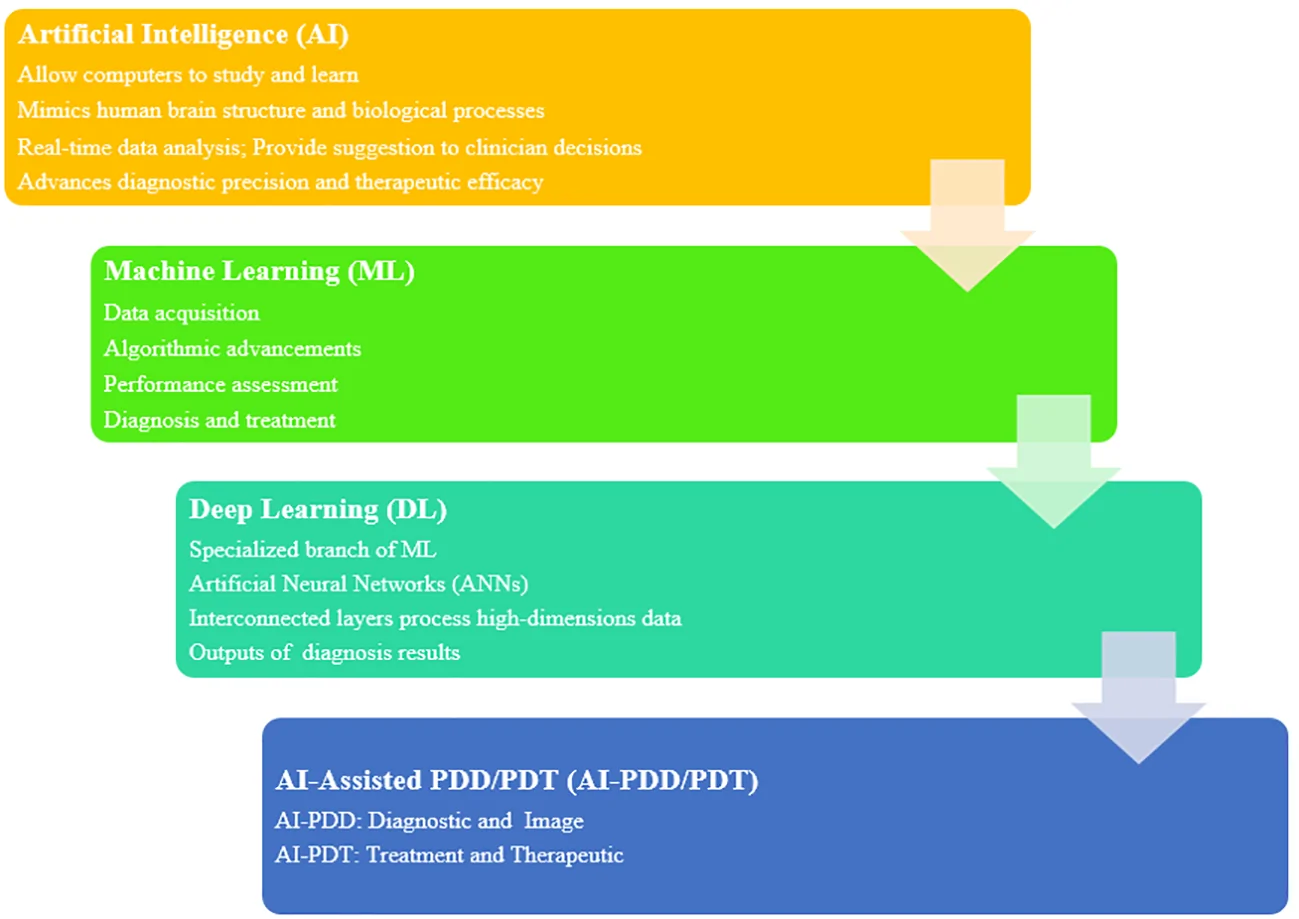
Flowchart for AI-assisted PDD/PDT (AI-PDD/PDT).

**Figure 1** outlines the operational domains where AI augments PDD/PDT, including explainable modeling, adaptive dosimetry, predictive outcome analysis, and automated image interpretation. This enables explainable modeling, adaptive dosimetry, predictive outcome analysis, and automated image interpretation. Meanwhile, AI can address current barriers of interpretability, reproducibility, and workflow integration (13). These capabilities support precision oncology by reducing diagnostic errors, guiding personalized therapies, and minimizing recurrence risks, such as data-driven approaches that use AI to transform PDD/PDT workflows.

Unlike prior studies that examine AI in oncology or photodynamic techniques in isolation, this review systematically evaluates the integration across diagnostic, therapeutic, and translational domains. This article aims to systematically evaluate how AI interacts with PDD/PDT across diagnostic, therapeutic, and translational domains. The novelty lies in its comparative and translational focus that highlights operational steps, advantages, recent progress, and future directions. By synthesizing evidence from preclinical, clinical, and computational studies, it provides a framework for advancing AI-assisted PDD/PDT toward routine clinical adoption.

## 2 Search strategy

This narrative review was conducted using search strings across major databases, including PubMed, Scopus, Web of Science, and the China National Knowledge Infrastructure (CNKI). The search covered publications from 2003 to 2025, guided by keywords such as *“*Artificial Intelligence” OR “Machine Learning” OR “Deep Learning” AND “Photodynamic Diagnosis” OR “Photodynamic Therapy OR “Cancer”. The study findings were presented and summarized without language restrictions (**Supplementary File 1**). Regarding the search strategy specifically targeted studies on bladder, gastric, colorectal, breast, and other cancers where AI-PDD/PDT has been applied for oncology.

Records were screened in two stages: (1) titles and abstracts were reviewed to exclude irrelevant studies, and (2) full texts were assessed for eligibility. Inclusion criteria comprised English-language studies that investigated AI-assisted PDD or PDT in oncology. Exclusion criteria included conference abstracts without full papers, non-oncology applications, and studies lacking methodological detail. The selection workflow identified 820 records: 240 from PubMed, 210 from Scopus, 190 from Web of Science, and 180 from CNKI. 150 duplicates were removed, and 670 records remained for screening. Title and abstract screening excluded 450 records as irrelevant. The remaining 220 full texts were assessed for eligibility, of which 164 were excluded, such as non-oncology focus, insufficient methodological detail, or conference abstracts only. Lastly, 56 studies were selected to fulfill the inclusion criteria and were synthesized in this narrative review (**Supplementary File 1**, **Supplementary Table 1**).

A narrative review approach was chosen because of the heterogeneity of evidence and the diverse domains represented in the literature on AI-assisted photodynamic diagnosis and therapy, spanning oncology, antimicrobial therapy, ophthalmology, and exploratory Chinese medicine. The literature highlights applications in tumor detection, surgical margin delineation, recurrence monitoring, and personalized PDT protocols within oncology. Furthermore, the emerging nature of the field means that many reports remain proof-of-concept, theoretical frameworks, or exploratory applications. Many of these reports remain at the proof-of-concept or preclinical stage, underscoring the need for translational studies and clinical validation in cancer research. Since the evidence base is not yet mature enough for quantitative synthesis or meta-analysis, a narrative review provides a comprehensive thematic overview of how AI integrates into PDD/PDT workflows, including diagnosis, dosimetry, predictive modeling, and translational opportunities. Therefore, this review emphasizes oncology as the primary domain, focusing on how AI-PDD/PDT contributes to precision cancer diagnosis, individualized therapy, and improved patient outcomes.

## 3 Overview of AI-PDD/PDT

The PDD process represents a critical component of clinical decision support systems, particularly those employing AI. Diagnostic errors remain prevalent, and the timely establishment of an accurate diagnosis is indispensable for the delivery of effective care. AI-driven algorithms are increasingly embedded within healthcare environments to augment clinician performance in diagnosis, therapeutic planning, and patient outcome prediction. ML and DL methodologies are designed algorithms to extract clinically relevant insights from complex datasets, thereby facilitating rapid and reliable diagnostic reasoning as well as evidence-informed treatment decision-making (14). This is particularly relevant for early cancer detection, tumor margin delineation, and recurrence monitoring, where AI-PDD reduces diagnostic errors and improves patient outcomes in oncology. The consequences of diagnostic error extend beyond clinical outcomes to encompass reputational, ethical, and moral dimensions for patients, clinicians, and healthcare organizations. Diagnostic errors can directly impact survival rates, treatment selection, and long-term prognosis, underscoring the importance of AI-driven accuracy for cancer patients.

**Figure 2:**
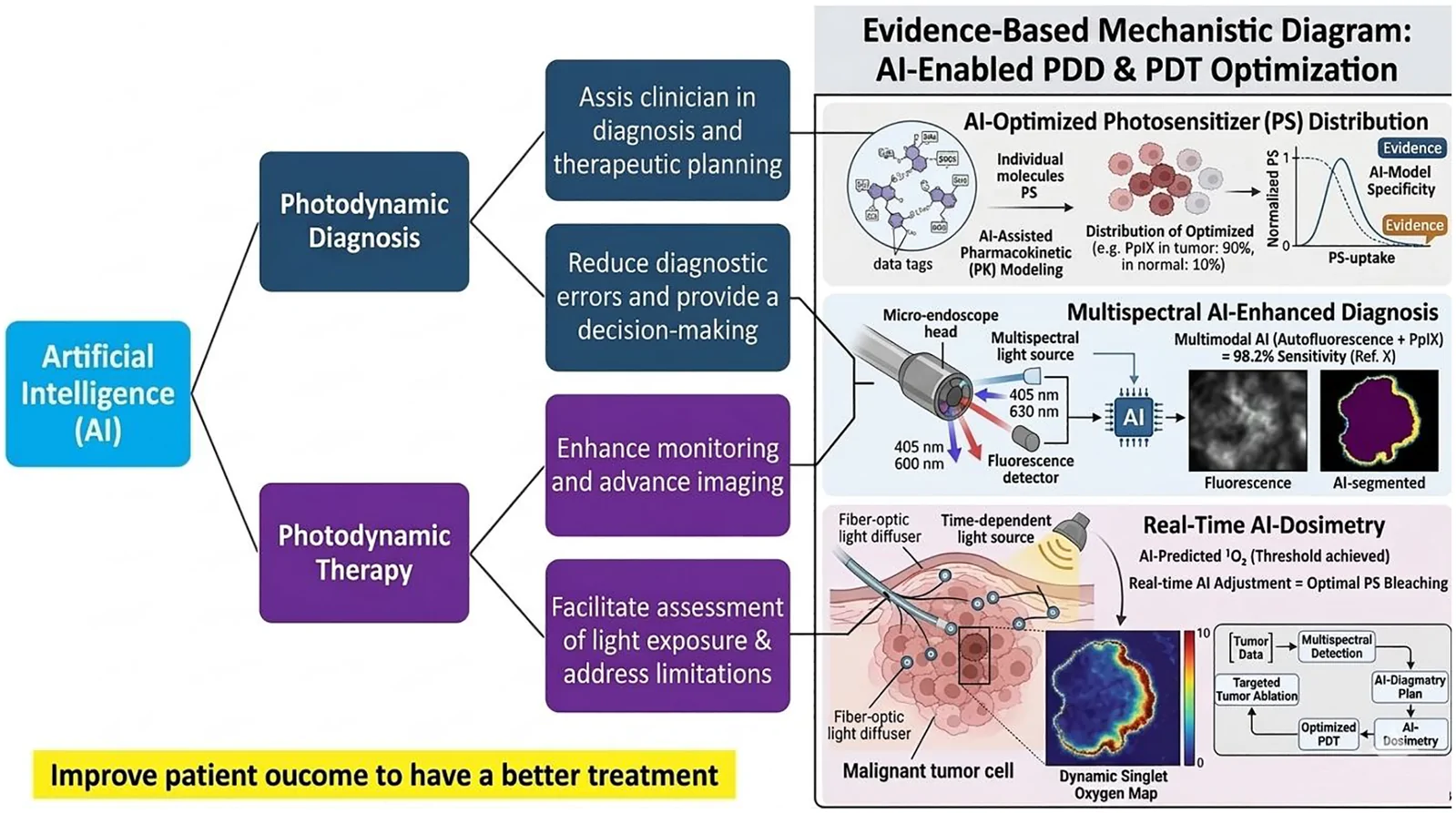
Evidence-based mechanistic diagram: AI-enabled PDD & PDT optimization.

Beyond PDD, AI has also been integrated with photodynamic therapy (AI-PDT). This integration enhances the precision and therapeutic efficacy of PDT by enabling real-time monitoring of treatment effects, advancing imaging modalities for improved diagnosis of specific diseases and tumor subtypes, and facilitating continuous assessment of light exposure during therapy (15). This enables precise dosimetry and real-time monitoring of tumor response, tailoring light delivery and photosensitizer activation to individual tumor biology in cancer therapy. Moreover, AI-PDT addresses key limitations in light delivery for deep-seated or internal tumors, thereby expanding the clinical applicability of PDT. It is especially critical in oncology, where deep-seated tumors such as colorectal, gastric, and breast cancers require optimized light penetration and photosensitizer distribution. Collectively, these advances hold significant potential to improve overall patient outcomes and strengthen the role of PDT within contemporary clinical practice (**Figure 2**) (16). Thus, AI-PDD/PDT represents a promising frontier in precision oncology, advancing cancer diagnosis, individualized therapy, and translational research.

## 4 AI-assisted PDD

AI-assisted PDD is an advanced diagnostic system. It integrates multimodal imaging, including fluorescence and ultrasonic modalities, across multi-centered and large-scale clinical networks. These networks enable multicenter cancer imaging studies, improving consistency in tumor detection and diagnostic workflows in oncology. For example, the combination of PDD and autofluorescence imaging (AFI) offers significant advantages over traditional white-light endoscopy in detecting precancerous lesions and early-stage cancers (17), which is particularly valuable in bladder, gastric, and peritoneal cancers, where AI-PDD enhances early detection and reduces missed lesions. This can achieve enhanced lesion recognition, automated fluorescence quantification, and improved diagnostic consistency. It translates into clearer tumor margin delineation, reduced false positives, and more reliable intraoperative guidance for cancer patients.

**Figure 3:**
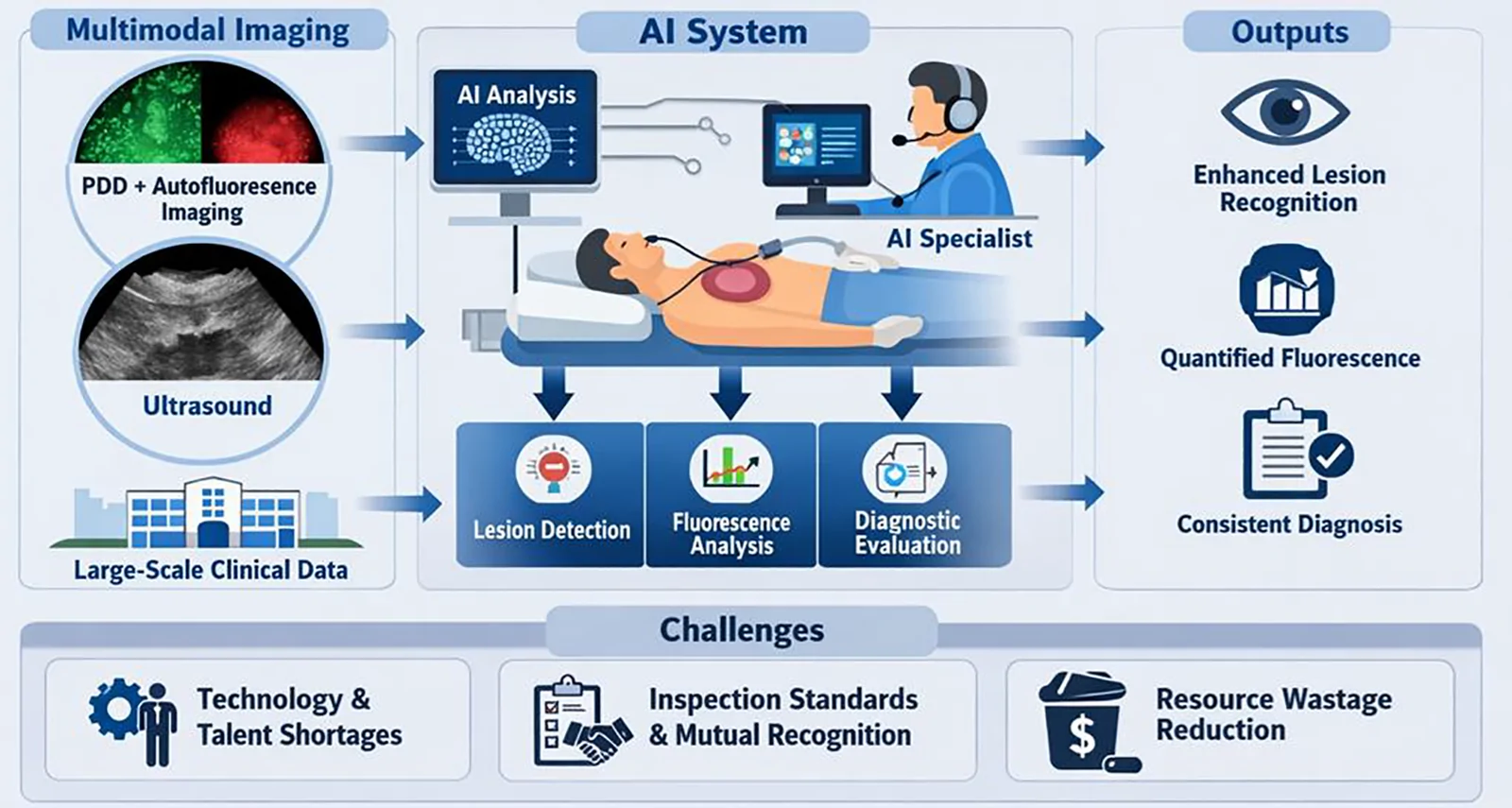
Overview of AI-assisted PDD for cancer.

However, AI integration with PDD depends on three major factors: (a) limiting technology and talent shortages, (b) establishing inspection standards and mutual recognition, and (c) reducing medical resource wastage (18):

- Enables faster and more accurate diagnosis and treatment planning within large volumes of data, but it lacks a specialist to operate and interpret the system to generate an image (19). This limitation can delay cancer diagnosis and treatment planning, highlighting the need for trained specialists to interpret AI-PDD outputs in oncology.
- Cystoscopy is the standard method for the early treatment of bladder cancer, but the mutual recognition may apply fluorescence to cystoscopy for detecting bladder cancer more accurately (20). Similar AI-PDD approaches are being explored for gastric and colorectal cancers, where fluorescence imaging can improve tumor visualization.
- Reduce retakes and repeated tests, halt without sufficient information to prevent waste (**Figure 3**) (21). It is preventing repeated imaging and unnecessary biopsies is critical for patient safety, cost efficiency, and timely treatment initiation in cancer care.

Convolutional neural networks (CNNs) and YOLO models are particularly suited for cystoscopic image analysis in PDD because they capture spatial hierarchies and enable real-time tumor detection. CNNs provide high sensitivity for subtle lesion recognition, while YOLO offers rapid detection for intraoperative use. However, these approaches demand large annotated datasets and are less interpretable than classical models. Their computational complexity requires graphics processing unit (GPU) support, which may limit adoption in smaller centers and increase training demands for healthcare workers. Interpretability can be partially improved through visualization methods such as gradient-weighted class activation mapping (Grad-CAM) (43, 52, 54, 56, 58).

ML algorithms applied to PDD, and CNNs currently demonstrate the strongest evidence for improving image segmentation, fluorescence quantification, and tumor margin delineation, thereby reducing false positives and enhancing diagnostic accuracy (14, 17). Support Vector Machines (SVMs) have been effective in smaller datasets, providing strong classification performance in early cancer detection tasks (18). Random Forest (RF) has shown advantages in handling structured clinical data, offering robustness and interpretability for predictive modeling of oxygen dynamics (32). Comparative advantages include CNNs excelling in high-dimensional imaging data, RF in structured tabular datasets, and SVMs in limited sample sizes (**Table 1**). These algorithms have complementary strengths for the advance AI-PDD workflows and improve diagnostic precision in oncology (14, 33).

**Table 1:** Comparative strength of ML algorithms in AI-PDD.

Algorithm	Strength	Limitation	Reference
Convolutional Neural Networks (CNNs)	High-dimensional imaging data; strong in tumor margin delineation, segmentation, and fluorescence quantification	Require large datasets; less interpretable	(14, 17)
Support Vector Machines (SVMs)	Strong classification in small datasets; early cancer detection	Limited scalability; performance drops with very large datasets	(18)
Random Forests (RF)	Robust with structured/tabular data; interpretable; effective in modeling oxygen dynamics	Less effective with complex imaging data	(32, 33)

## 5 AI-assisted PDT

The operation of AI-assisted PDT is categorized into three levels: (i) treatment workflow for regulating photosensitizer (PS) administration and light activation for timing; (ii) dosimetry and real-time monitoring for optimizing light parameters (wavelength, intensity, pulse duration), modeling oxygen concentration and reactive oxygen species (ROS) dynamics, and ensuring uniform light delivery; and (iii) outcome prediction for integrating PS characteristics, patient data, and treatment parameters into machine learning models to forecast therapeutic efficacy. These levels are critical for tailoring PDT protocols to specific tumor types, improving precision in cancer treatment in oncology. The variables requiring optimization include PS type and dose, light source parameters, oxygen availability, ROS generation, and timing of PS administration relative to light exposure.

- In general, the treatment workflow comprises PS administration, biodistribution and accumulation, light activation, cellular or tissue responses, and post-treatment care. The administration of PS is delivered via intravenous or topical application. The PS is circulated by the bloodstream and selectively accumulated in target tissues for around 15 to 20 mins. Subsequently, light irradiation is used to activate the PS with a specific wavelength, such as a laser, light-emitting diode (LED), or fiber-optic probes. Upon activation, the PS generates reactive oxygen species (ROS), which induce cytotoxic effects leading to damage of abnormal vessels or tissues. Patients are advised to take rest and avoid exposure to strong light for 24 to 48 hours to minimize photosensitivity risks after treatment (22). This workflow has been applied to bladder, gastric, breast, and colorectal tumors, where AI-assisted PDT enhances cytotoxicity against malignant cells while sparing healthy tissue in cancer therapy.
- AI optimizes the treatment workflow through regulating the injection volume and light activation timing, changing and improving dosimetry, as well as automating workflow decisions. It integrates patient-specific data to determine the best PS administration, light delivery parameters, and post-treatment precautions. AI-driven robotic compounding systems have been employed in hospital pharmacies for intravenous drug preparation and can be adapted to standardize PS infusion preparation. These platforms automate dosing, dilution, and mixing under sterile conditions, thereby reducing variability and minimizing human error, which may lead to incorrect dosing, contamination, or inconsistent preparation in manual practice that compromises PDT efficacy and patient safety (23). The selective targeting of PS accumulation is primarily determined by intrinsic tissue properties. AI contributes indirectly by customizing PS choice and dosage according to patient-specific tumor characteristics. Furthermore, AI systems can integrate variables such as PS absorption and emission spectra, tissue scattering and penetration depth, oxygen availability, and ROS dynamics to optimize light parameters including wavelength, pulse duration, and intensity, ensuring effective activation of PS while minimizing off-target damage (24). This customization is particularly important in oncology, where tumor heterogeneity requires individualized PS selection and light delivery strategies to maximize therapeutic benefit.

Thus, the critical parameters of dosimetry include the light source, type of PS, and dosage. Because AI can be leveraged to explore and optimize optical properties for different wavelengths to identify the most suitable conditions of PS activation. It may choose the specific wavelength, pulse duration, and light intensity for the excitation of PS to reduce tissue damage. The ML models of AI propose the absorption, emission, photostability, and photobleaching, tissue penetration, as well as the rational design of PS for targeting (25). Basically, AI-driven physiologically based pharmacokinetic/pharmacodynamic (PBPK/PD) modeling measures and simulates PS distribution, clearance, and therapeutic window to ensure its safety and efficacy. This modeling framework provides predictions of PK and PD outcomes for the target. It guides the rational treatment design at an early discovery stage. The integrated platform for ML algorithms further enhances predictive capacity by linking PK profiles to PD effects. This alignment optimizes the PS pharmacokinetics and pharmacodynamic responses to ensure the PDT protocols, which are tailored for maximal therapeutic benefit from the outset (26). PBPK/PD modeling has been used to predict photosensitizer distribution in solid tumors, guiding safe and effective PDT dosing in cancer research.

Meanwhile, AI facilitates the development of navigation systems that ensure uniform light delivery during the PDT process (27). It integrates all of the spectral data for multiple tissues to predict their scattering, absorption, emission, fluorescence, and penetration depth. This allows the selection of wavelengths to balance therapeutic efficacy with minimal off-target effects. The role of AI is improving data collection, automating data analysis, extracting meaningful insights, and enabling predictive modeling in PDT (28), which ensures uniform light delivery across irregular tumor surfaces, improving treatment consistency and reducing recurrence risks for oncology.

AI can also enable the automation of workflow decisions by designing the integration of PDT platforms, which apply to Quantitative Clinical Pharmacology (QCP) and Translational Sciences (TS). These platforms operate autonomously at various levels, where specialized AI modules are chosen to collaborate with PDT systems in complex tasks. The processes, such as data collection, analysis, modeling, and simulation, are AI-driven workflows to enhance their efficiency, reproducibility, and consistency in clinical decision-making (29). These AI-enabled platforms are the therapeutic development pipeline from PS discovery to late-stage clinical stages (30). It also platforms accelerate PS discovery and translation into clinical oncology trials.

Besides, PDT efficacy depends on the measurement of oxygen and ROS generation. Oxygen is a fundamental substrate for the generation of ROS. When oxygen availability is limited, the efficiency of PDT is markedly reduced (31). AI approaches employ machine learning algorithms such as RF, which aggregates multiple decision trees to improve predictive accuracy, and multilayer perceptron (MLP), a neural network architecture capable of modeling nonlinear relationships. To reduce data dimensionality, wrapper feature selection methods iteratively evaluate subsets of variables to identify the most relevant features for prediction. Together, these approaches have been applied to modeling oxygen volume during the PDT process, enabling more precise estimation of treatment efficacy (32). Modeling oxygen and ROS dynamics is essential for predicting tumor response, as hypoxic microenvironments often limit PDT effectiveness in oncology. ML in AI emerged as a powerful tool to model ROS dynamics, predict cytotoxic outcomes, and optimize PS design. There is a relationship between the level of ROS and cytotoxic outcomes to predict the efficacy of PDT (33).

**Figure 4:**
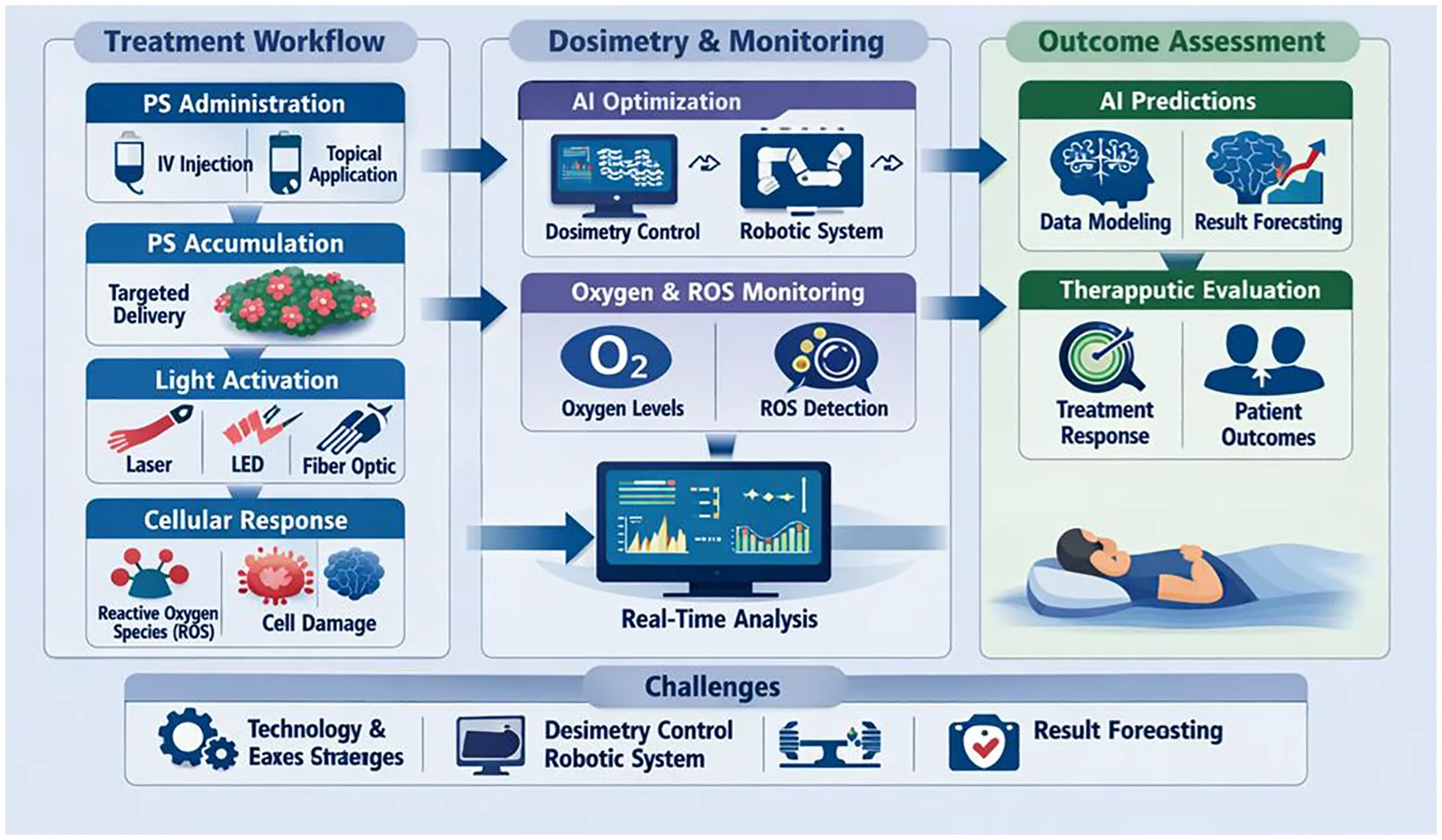
Overview of AI-assisted PDT for cancer.

(c) AI can assess outcomes and predict the results by integrating specific data, modeling oxygen or ROS dynamics, which applies ML to forecast therapeutic efficacy. AI-based predictive analytics employs advanced algorithms and ML techniques to analyze large-scale datasets encompassing injection parameters, characteristics of PS, and the outcomes of PDT treatment. AI systems generate predictive models capable of forecasting therapeutic responses with greater precision than conventional methods by identifying underlying patterns and correlations within the analyzed data. Moreover, the AI framework continuously receives, learns, and adapts from data. It enables iterative refinement and progressive improvement in outcome prediction over time (34). Oxygen model and ROS dynamics involve the development of computational frameworks through minimalist to detailed mechanistic models for simulating the generation of ROS and elucidating the roles of cellular function. Reaction and diffusion equations and molecular dynamics are employed to explore phenomena spanning cellular respiration that cause oxidative damage to provide mechanistic insight into PDT treatment (**Figure 4**) (35). Therefore, AI-assisted PDT represents a promising frontier in precision oncology, enabling individualized cancer therapy and advancing translational research (**Table 2**).

**Table 2:** AI-assisted PDT operation.

Process	Components	AI-integrations	Clinical and research implications
(a) Treatment workflow	(1) PS administration, biodistribution, and accumulation (2) Light activation by laser, LED, fiber optic (3) ROS generation and cytotoxicity (4) Post-treatment care	(1) & (2) Optimized injection time and PS light interval, automate workflow decisions (3) & (4) Driven robotic systems for PS preparation, customize PS selection and dosage based on tumor characteristics	Enhance reproducibility and reduce human error, improve precision in targeting cancers
(b) Dosimetry and Real-time monitoring	(1) Critical parameters: light source, PS type, dosage (2) Optical properties: wavelength, pulse duration, intensity (3) Oxygen and ROS measurement	(1) & (2) AI explored optical conditions via ML model, PBPK/PD model, and navigation system (3) Predictive model for oxygen or ROS dynamics	(1) & (2) Tailored PDT protocols and guidelines, balance efficacy based on off-target effects, enhance efficiency, and consistency in the clinical treatment
(c) Outcome assessment and Prediction	(1) Integration of injection parameters, PS characteristics (2) Mechanistic frameworks for oxygen or ROS dynamics	(1) ML algorithms identified patterns, correlations, and continuous learning for iterative refinement (2) Forecasts cytotoxic outcomes based on ROS levels	(1) More precise outcome prediction than conventional methods (2) Progressive improvement in personalized treatment

ANNs are flexible for modeling nonlinear biological responses, such as PS uptake, but risk overfitting and require validation. RF models excel with structured dosimetry data, offering interpretability and robustness against noise. Compared with CNNs, these methods are computationally lighter and more accessible for smaller datasets. The clinical applicability lies in treatment planning and dosimetry prediction, and the need for external validation (32, 33, 47, 50, 61).

## 6 Advantages of AI-PDD/PDT for cancer

AI-PDD improves imaging accuracy and enhances diagnostic performance by applying deep learning algorithms to multimodal imaging data including fluorescence, autofluorescence, and white-light signals. These algorithms improve image segmentation, fluorescence quantification, and lesion classification, thereby reducing observer variability, strengthening tumor fluorescence visualization, lowering false-positive rates, and facilitating early cancer detection (14). This capability is crucial for identifying early bladder, gastric, and peritoneal cancers, improving patient prognosis through timely intervention in oncology. It supports the screening of primary mammary tumor tissues, consistently demonstrating significantly higher fluorescence intensity compared to surrounding normal mammary tissue. This distinction enables clear intraoperative delineation of tumor margins, thereby improving diagnostic accuracy and guiding precise surgical excision (36). The intraoperative guidance is particularly valuable in breast cancer surgery, where accurate margin delineation reduces recurrence risk.

The clinical advantages of AI-PDT usage include real-time monitoring, precise dosimetry, and improved outcome prediction. More critically, AI enables personalized treatment planning in PDT. This involves integrating patient medical history with knowledge of the spatial and temporal co-localization of photons, PS, and oxygen. It is an individualized approach to address the significance of inter- or intra-patient variability of the PS concentration, and often results in suboptimal outcomes when relying on standardized treatment protocols (37). This individualized planning ensures optimal photosensitizer dosing and light delivery tailored to tumor biology, enhancing therapeutic efficacy in cancer therapy.

AI-PDT represents a promising avenue for advancing research, particularly in the accelerated benchmarking of PS. AI can rapidly evaluate both natural and synthetic PS, predicting their biodistribution, cellular uptake, and phototoxicity profiles with greater accuracy and efficiency through data-driven analysis. Because AI not only advances personalized medicine but also enhances predictions of absorption, distribution, metabolism, excretion, and toxicity (ADMET), while simultaneously optimizing PS structures through predictive modeling and rational design strategies (38). These strategies accelerate the discovery of novel photosensitizers with improved tumor selectivity and reduced toxicity for oncology. The strategies increase the theranostic potential of PDT, as its efficacy depends on some key parameters, including the localization and concentration of PSs at the tumor site, the administered light dose and intensity, oxygen availability, ROS generation, and the heterogeneity of the tumor microenvironment, which are optimized by advanced imaging techniques (39). This theranostic integration supports precision oncology by combining diagnostic imaging with targeted therapy, ultimately improving survival outcomes in cancer care.

AI-PDD/PDT show promise across imaging, dosimetry, and predictive modeling, and it is important to distinguish between technologies that have reached clinical validation and those that remain at the proof-of-concept stage. For example, bladder cancer PDD using 5-ALA cystoscopy has demonstrated consistent clinical benefit in reducing recurrence and progression rates in national cohorts with a validated application (8, 9), which represents the only consistently validated clinical application to date. In contrast, many AI-driven approaches, such as predictive modeling of ROS, PBPK/PD simulations of PS distribution, and lysosome-targeted PSs, remain at preclinical or exploratory stages (12, 26, 33). These proof-of-concept studies highlight future potential but require systematic validation through multicenter trials before integration into oncology guidelines. Such clear separation of validated versus experimental evidence is essential to avoid overstating readiness and to guide translational research priorities (14, 34).

## 7 Research process

AI-PDD is a precise technique in which a photosensitizing agent preferentially accumulates in malignant tumor cells and pathological vasculature, thereby enhancing fluorescence visualization of diseased tissues while sparing surrounding healthy cells. Upon activation with specific wavelengths of light, these agents emit fluorescence, making tumors visible for early detection, mapping, and surgical guidance (40). This approach has been applied to bladder, gastric, and peritoneal cancers, improving early detection and guiding surgical excision with enhanced tumor margin visualization in oncology. Based on the above advantages of AI-PDD/PDT, many scientists have paid attention to the current research within the past five to ten years.

The AI-PDT research and applications provide a platform for enhancing treatment precision, PS discovery, and clinical outcome prediction for Western medicine. AI-PDT has demonstrated potential to personalize treatment protocols, optimize photosensitizer dosing, and predict tumor response more accurately in cancer therapy. These studies collectively demonstrate the usage of ML and DL approaches that are reshaping PDT across antimicrobial, oncological, and ophthalmological domains. Current research has been extended to the conceptual, preclinical, and translational stages, with oncology emerging as the primary domain for PDT applications. This dominance reflects the urgent need for AI-driven innovations in cancer care, where improved imaging, photosensitizer discovery, and treatment evaluation directly impact survival outcomes. DL is driving advances in imaging, PS discovery, and treatment evaluation in the development of AI-PDD/PDT for Western medicines in **Table 3**.

**Table 3:** Research process of AI-assisted PDD (1–4)/PDT (5–11).

	Topic	AI integration	PS	Outcome	Sample size	Validation strategy	Performance metric	Study limitation	Clinical maturity	Reference
1	AI-guided laparoscopy in rabbit peritoneal carcinomatosis (PDD)	Real-time AI algorithm screened nodules	Phonozen	Improved detection of metastatic peritoneal cancer	270 nodules (rabbit model)	Internal validation	Sensitivity ↑ to 100%	Preclinical animal model only	Preclinical	(41)
2	5-ALA endoscopic PDD for gastric tumors (PDD)	LAB color mapping & CNN	5ALA	Enhanced tumor identification	3030 images (clinical dataset)	Cross-validation	Accuracy ~90%, AUC reported	Single-center, retrospective	Early clinical	(42)
3	Detecting false positives in PDD (PDD)	Deep learning	Not specified	Improved discrimination between true vs. false positives	Small dataset (urology cases)	Leave-one-case-out	Sensitivity/specificity reported	Limited sample, pilot study	Early clinical	(43)
4	Real-time AI-guided laparoscopy in rabbit metastasis (PDD)	YOLOv8 neural network	Phonozen	Sensitivity ↑ to 100%	Rabbit model (n≈20)	Internal validation	Sensitivity 100%	Preclinical only	Preclinical	(44)
5	AI framework for PDT antimicrobial effectiveness (PDT)	AI framework for PS/light parameter selection	None	Conceptual foundation	None (conceptual)	Not applicable	Not applicable	No experimental validation	Conceptual	(45)
6	AI-enabled implantable telemetry for PDT (PDT)	DeepLabCut & thermal/light simulation	Hypericin, Foscan	Optimized illumination, suppressed xenograft growth	Xenograft mice (n≈20–30)	Internal validation	Tumor suppression rates	Small animal study	Preclinical	(46)
7	Data-driven discovery of NIR type I PSs (PDT)	Deep learning screening pipeline	Type I PSs	Identified novel NIR candidates	Large compound library	Computational validation	AUC >0.9 for screening	No clinical validation	Computational discovery	(47)
8	DL insights into PDT effects on cancer cells (PDT)	Cellpose algorithm	AuNP@Tetramethylthazine	Predicted PDT effects at single-cell level	Cell culture images	Internal validation	Accuracy >85%	*In vitro* only	Preclinical (*in vitro*)	(48)
9	ML-assisted cyanine PS design (PDT)	ML molecular modeling	Cyanine derivatives	Rational design of theranostic agents	Computational dataset	Model validation	AUC/ROC reported	No clinical validation	Computational discovery	(49)
10	DL evaluation of PDT on tumors (PDT)	YOLOv3 cell detection	ICG-Ce6 nanoparticles	Quantified live/dead cells post-PDT	Tumor cell datasets	Internal validation	Accuracy ~92%	*In vitro*, limited scope	Preclinical (*in vitro*)	(50)
11	DeepPDT-Net for CSC chorioretinopathy (PDT)	ResNet50 transfer learning multimodal	None	Improved prediction of treatability	18689 images (clinical dataset)	Two-stage multimodal validation	AUC ~0.85	Retrospective, single-center	Early clinical	(51)

Based on the findings, a comparative **Table 4** is designed and provides information about how AI has been applied for PDD/PDT. These applications span bladder, gastric, colorectal, breast, and peritoneal cancers, highlighting AI’s role in tumor detection, margin delineation, and therapeutic optimization in oncology that organizes the application area, data modality, AI approach, dataset size, validation strategy, outcomes, limitations, and stage of translation for the distinctions between conceptual proposals, preclinical proof-of-concepts, retrospective analyses, and early clinical validations. The comparative framework underscores that most AI-PDD/PDT evidence in oncology remains at the conceptual or preclinical stage, with limited but promising early clinical trials.

**Table 4:** Compared to the AI-PDD/PDT studies approach.

Application	Data modality	AI approach	Dataset size	Validation	Outcomes	Sample size	Validation strategy	Performance metric	Limitation	Stage of translation	Reference
PDD Peritoneal Cancer	Endoscopic video (rabbit model)	YOLOv8	270 nodules	Real-time monitoring	100% sensitivity in metastasis detection	270 nodules (animal)	Internal validation	Sensitivity ↑ to 100%	Animal model only, limited generalizability	Preclinical (animal study)	(41, 44)
PDD Gastric Tumor	Endoscopic fluorescence images	CNN	500 images	Cross-validation	Improved tumor vs. non-tumor classification	500 images (clinical dataset)	Cross-validation	Accuracy ~90%, AUC reported	Small dataset, single-center	Early clinical (retrospective dataset)	(42)
PDD False Positive Reduction	Bladder cancer images	Deep learning	200 cases	Leave-one-case-out	Enhanced discrimination of true vs. false positives	200 cases	Leave-one-case-out	Sensitivity/specificity reported	Small dataset, site-specific bias	Early clinical (retrospective dataset)	(43)
PDT Antimicrobial Identification	Microbial profiles	Predictive modeling	Not specified	None (conceptual)	Framework for personalized PDT	None	Not applicable	Not applicable	No experimental validation	Conceptual (algorithm only)	(45)
PDT Tumor Illumination	Xenograft models	DeepLabCut & telemetry	Not specified	Simulation experiment	Optimized multi-wavelength illumination, tumor suppression	Xenograft mice (n≈20–30)	Internal validation	Tumor suppression rates	Limited to animal models	Preclinical (animal study)	(46)
PDT PS Discovery	Molecular datasets	ML-assisted design	Large chemical libraries	Experimental validation	Identified novel cyanine-based PS	Large compound library	Computational and experimental validation	AUC/ROC reported	Synthetic feasibility not tested	Discovery/Optimization (molecular design)	(47, 49)
PDT Dynamic Cell Effects	Cell imaging	Cellpose algorithm	Cell-level dataset	Algorithm validation	Accurate monitoring of PDT effects	Cell culture images	Internal validation	Accuracy >85%	*In vitro* only, no clinical validation	Preclinical (*in vitro* only)	(48)

### 7.1 Complementary pathways in AI-PDD/PDT against cancer

AI-PDD/PDT has evolved along two complementary strategies, including standardized biomedical pathways and natural compound-oriented frameworks. These complementary strategies provide distinct but synergistic approaches to advancing cancer diagnosis and therapy in oncology.

Standardized biomedical pathways emphasize rigorous clinical conditions, evidence-based protocols, and regulatory requirements. These approaches are particularly advanced in oncology, with established applications in bladder, gastric, and peritoneal cancers, focusing on the use of chemically synthesized photosensitizers, ensuring high imaging accuracy, reproducibility, and consistency across multicenter clinical trials (52). The trials demonstrate how AI-PDD/PDT improves diagnostic accuracy and therapeutic reproducibility in cancers such as bladder, gastric, colorectal, and breast tumors.

On the other hand, natural compound-oriented frameworks integrate AI technologies with photosensitizers derived from flavonoids, porphyrins, and herbal extracts, while adhering to holistic diagnostic principles. AI can be used for compound screening by analyzing large datasets of natural molecules to predict fluorescence properties, biocompatibility, and therapeutic potential, which enables prioritization of promising photosensitizers for experimental testing, thereby accelerating discovery while adhering to holistic diagnostic principles (53). It expands the repertoire of photosensitizers by identifying natural compounds with tumor-selective fluorescence and cytotoxicity, offering novel therapeutic options for oncology.

These pathways are complementary: standardized biomedical approaches advance clinical validation and regulatory translation, whereas natural-compound strategies contribute novel agents and individualized therapeutic frameworks. The synergy highlights the potential of AI-PDD/PDT to bridge biomedical rigor with personalized, nature-inspired innovation, and improve cancer care.

### 7.2 Specific examples for AI-PDD on cancer

Several studies have demonstrated the application of AI-PDD specifically in oncology, with bladder cancer serving as a primary model for clinical validation. Nairveen et al. reported the use of deep learning–based classification of blue light cystoscopy imaging for transurethral resection of bladder tumors (54). PDD has enabled the detection of flat and small lesions for improving visualization of malignant versus benign tissues. While PDD itself does not directly determine malignancy, invasiveness, or grading. AI-enhanced approaches, such as brain-inspired deep neural networks (DNNs), can analyze fluorescence patterns and lesion morphology to support classification tasks. These DNNs have achieved state-of-the-art performance in object recognition, approaching human-level accuracy in categorization (55).

Similarly, Mutaguchi et al. demonstrated the utility of AI using a dilated U-Net architecture, which expands the receptive field of the network to capture both fine detail and broader context. This approach reduced the risk of overlooked bladder tumors compared with conventional AI segmentation systems (56). This approach improved diagnostic accuracy and addressed the issue of poor reproducibility, which often arises from dependence on physician experience and subjective interpretation (57).

Ikeda et al. further indicated that cystoscopic images analyzed with convolutional neural networks (CNNs) could be effectively classified into tumor lesions and normal tissue (58). Endoscopic techniques such as narrow band imaging (NBI) and photodynamic diagnosis (PDD) were thus developed to enhance visibility and improve therapeutic outcomes in bladder cancer management (59).

### 7.3 Specific examples for AI-PDT on cancer

Several studies have demonstrated how AI-PDT is applied in oncology, with solid tumors serving as primary models for computational prediction and clinical validation. Tejedor-Estrada et al. developed an ANN model to predict the subcellular localization of photosensitizers in solid tumors. This computational approach analyzes molecular features to forecast whether PSs accumulate in organelles such as mitochondria or lysosomes, thereby informing their potential therapeutic effectiveness. There were a total of 128 two-dimensional molecular descriptors encompassing lipophilicity or hydrophilicity, charge, and structural characteristics initially calculated. These were reduced to 76 through Pearson’s correlation coefficient analysis, and subsequently narrowed to 5 using the feature selection algorithm proposed by Guyon and Elisseeff. Localization data for 61 PSs were compiled from the literature and categorized into three cellular compartments: mitochondria, lysosomes, and other organelles. The main purpose was to address the PS and organelle assignment problems for predicting the subcellular localization of PSs. To achieve this, a non-linear ANN was implemented within a decision-tree framework, enabling the distinction between different organelle localizations and thereby informing PDT efficacy in solid tumors (60). This approach highlights how AI can guide photosensitizer selection and localization to improve therapeutic outcomes in cancer treatment.

Yassine et al. also reported that ML for real-time optical property recovery in interstitial PDT increased the robustness of PDT against optical properties variations by using ML models to recover the patient-specific light dosimetry measurements and reduced uncertainty in the predicted light dosimetry for all tissues involved (61). This ensures robust dosimetry in heterogeneous tumor environments, reducing uncertainty in light delivery in oncology.

He et al. identified that AI-assisted DNA aneuploidy cytology for surveilling dysplastic oral leukoplakia treated by ALA-PDT, which surveilled non-homogeneous oral leukoplakia with moderate-to-severe dysplasia. The scalpel biopsy remained a reliable method for monitoring the prognosis of oral leukoplakia (62). It demonstrates AI-PDT’s potential in managing precancerous lesions and preventing malignant transformation.

Algorithm reflects task requirements, such as CNNs and YOLO dominating imaging-based detection (42, 43, 54, 56), while RF and ANNs are favored for modeling oxygen dynamics and PS localization (32, 33, 60). CNNs achieve superior accuracy but lack transparency, YOLO prioritizes speed at the expense of sensitivity, and RF offer interpretability for weaker performance in high-dimensional imaging. These trade-offs must align algorithm selection with clinical context, dataset size, and the need for interpretability (40–62).

## 8 Critical perspectives on AI-PDD/PDT

The evidence base for AI-PDD/PDT remains heterogeneous, spanning feasibility studies to measure clinical trials. PDD has been evaluated in urothelial malignancies, including upper urinary tract transitional cell carcinoma (40) and bladder cancer in multicenter randomized trials of fluorescence cystoscopy (52). These investigations demonstrated improved tumor detection, but concerns persist regarding false positives and reproducibility. DL applications to cystoscopic imaging, such as classification (54), segmentation (56), and diagnostic support systems (58). They have achieved promising accuracy, and most remain single-center studies without external validation. Endoscopic AI-PDD for gastric tumors (42) and approaches to reduce false positives (43) illustrate technical progress, and they underscore the need for standardized evaluation metrics. Preclinical work in peritoneal carcinomatosis models further highlights the potential of AI-guided laparoscopy to enhance sensitivity (44).

AI has been leveraged for PS discovery (47, 49, 60) in PDT, dosimetry optimization (50, 61), and cytology surveillance (62). These advances demonstrate strong preclinical promise but limited clinical translation. For example, DL models for treatment evaluation (50) and optical property recovery (61) remain simulation-based or small-scale, while AI-assisted cytology has shown utility in monitoring oral leukoplakia treated with PDT (62). Conflicting evidence persists regarding whether AI integration meaningfully reduces recurrence rates or primarily enhances detection sensitivity (40, 52). There were many investigations with methodological limitations, including small sample sizes and lack of multicenter validation, conflicting outcomes like sensitivity versus specificity trade-offs, and a translational gap between preclinical innovation and clinical oncology practice (40–62). Future research should prioritize multicenter trials, standardized AI reporting, and integration with multimodal therapies to ensure robust and clinical adoption of AI-PDD/PDT.

## 9 Discussion

**Figure 5:**
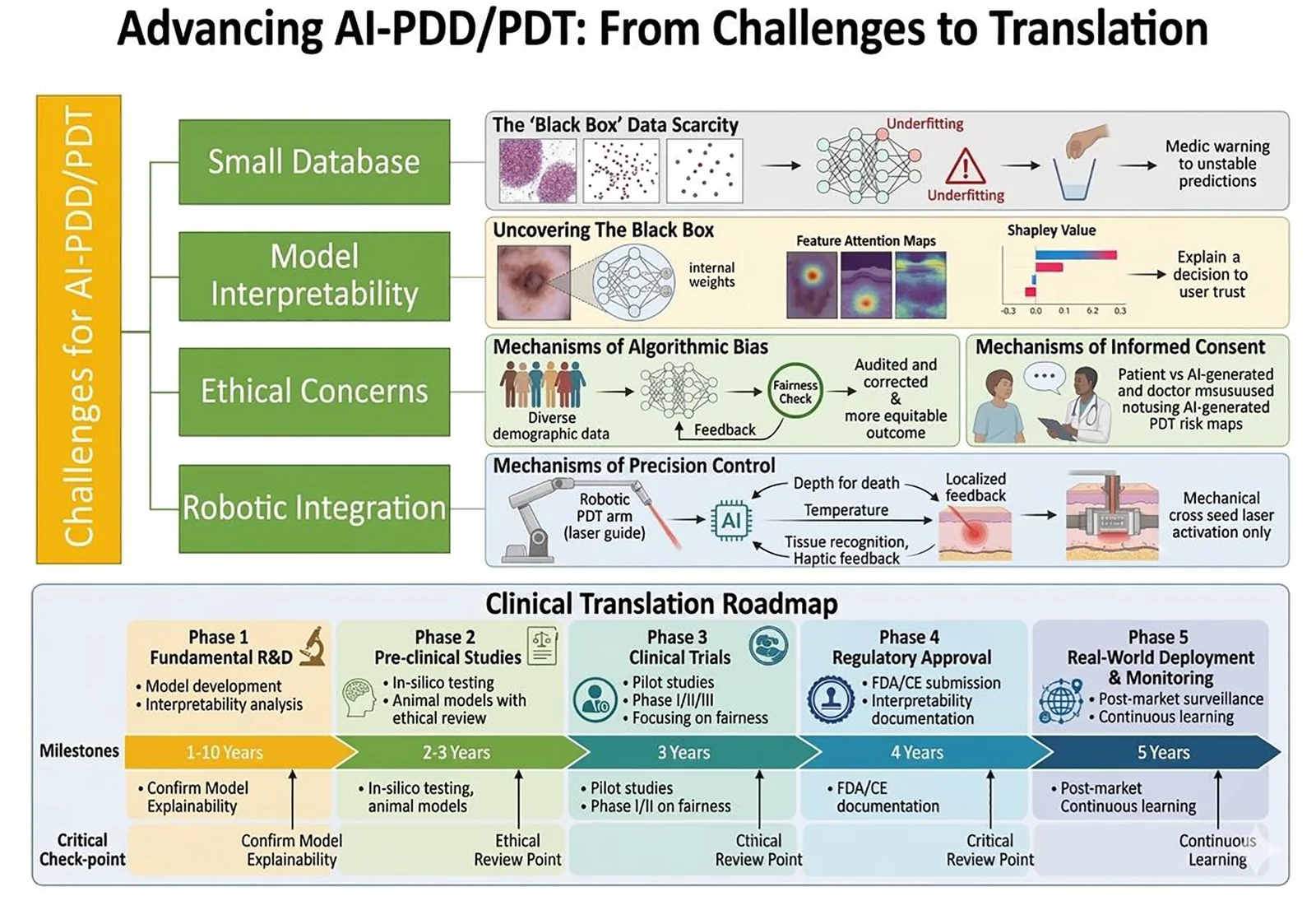
Advancing AI-PDD/PDT: from challenges to translation.

AI has emerged as a transformative force across medicine and the life sciences in recent years. Its applications demonstrate immense potential, ranging from the timely detection of pandemic disease outbreaks to advances in radiological and histological diagnostics that are reshaping conventional healthcare practices, according to the above information. These advances are particularly transformative, enabling earlier cancer detection, improved tumor classification, and more precise therapeutic planning in oncology. The integration of AI into PDT and multi-omics investigations provides powerful tools for characterizing complex molecular profiles, thereby deepening our understanding of disease mechanisms and enabling more personalized therapeutic strategies (63). Multi-omics integration with AI-PDT provides insights into tumor heterogeneity, guiding individualized treatment protocols and predicting therapeutic response for cancer care. AI has been increasingly explored in PDT, supporting PS discovery and dosimetry optimization in preclinical and early exploratory studies. Evidence of improved survival outcomes remains preliminary and requires validation in larger clinical trials. However, a series of questions needs to be considered (**Figure 5**). These questions are especially critical in cancer research, where translational maturity remains limited and rigorous validation is required to move AI-PDD/PDT from conceptual promise to clinical oncology practice.

### 9.1 Advantages and specificity of AI-PDT for cancer

AI-PDT has the potential to enhance treatment precision, enable patient-specific personalization, and address workflow challenges. This strengthens the dosimetry and automates critical clinical decisions by optimizing light synchronization (64). It synchronization is critical for maximizing tumor cytotoxicity while minimizing collateral damage to surrounding healthy tissues in oncology. The ML and DL techniques in AI support the identification and summarization of relevant information as well as the calculation of statistical parameters, such as light, tissue oxygenation, and inherent properties of the PSs.

Many operational limitations of conventional PDT alone have been addressed in AI-PDT research and practice (**Table 5**). ML and DL provide computational frameworks that manage the complexity of PDT variables, accelerate PS discovery, optimize treatment planning, and enable patient-specific outcome prediction. Its tailoring PDT protocols to tumor heterogeneity, improving reproducibility, and reducing recurrence risks for cancer therapy. Conventional PDT uses a fixed, manually chosen wavelength, whereas AI-PDT employs adaptive wavelength adjustment in real time to optimize treatment. Similarly, conventional PDT relies on direct ROS measurement, while AI-PDT predicts ROS dynamics by modeling their generation and clearance, enabling proactive control of therapeutic outcomes.

**Table 5:** Comparative analysis for traditional PDT and AI-PDT.

Operation step	Conventional PDT	AI-PDT	Validation strategy	Performance metric	Limitation	Stage of translation
PS Injection/Administration	Human handling, risk of error	Robotic infusion, reproducibility	Preclinical/animal validation	Error reduction rates	Limit clinical adoption, requires robotic infrastructure	Preclinical–Early clinical
Light Activation	Fixed, manually chosen wavelength	Adaptive wavelength adjustment	Simulation and preclinical validation	Sensitivity/specificity of tumor detection	Limit external validation, hardware complexity	Preclinical
Dosimetry	Empirical, trial-and-error	PBPK/PD modeling, ML optimization	Computational and retrospective datasets	AUC >0.85	Mostly computational, limit clinical benchmarking	Discovery–Preclinical
ROS Monitoring	Direct ROS measurement	ROS dynamics prediction via ML	*In vitro* and computational	Accuracy ~85–90%	No standardize clinical ROS monitoring	Preclinical (*in vitro*)
Outcome/Effectiveness	Retrospective analysis	Iterative learning, predictive analytics	Retrospective and pilot studies	Improve sensitivity/specificity in small datasets	Few multicenter prospective trials	Early clinical (limited)

Conventional PDT has theoretical and mechanistic limitations that depend on three factors: (a) light, (b) tissue oxygenation, and (c) inherent properties of the photosensitizer (65). These fundamental determinants remain regardless of the method of data analysis. While AI-PDT cannot alter these intrinsic factors, it can overcome practical limitations by optimizing light delivery, modeling oxygen consumption and ROS dynamics, and assisting in the discovery of improved photosensitizers. Thus, AI-PDT enhances the precision and adaptability of PDT within the boundaries set by these biological and physical constraints. These practical limitations is overcoming for translating PDT into reliable cancer therapies, particularly for solid tumors with complex microenvironments in oncology.

a. Light activates the photosensitizer in PDT, triggering the formation of ROS. These ROS initiate peroxidative reactions that compromise cellular membranes, proteins, and nucleic acids, leading to vascular shutdown, apoptosis, or necrosis as the major cytotoxic effects (66). These mechanisms are particularly relevant in cancer therapy, where selective vascular shutdown and apoptosis contribute to tumor eradication. Oxygenation levels in the surrounding treatment area can influence PDT efficacy, but monitoring them before and after treatment is not routine clinical practice and remains largely experimental. Johansson et al. reported that there was a real-time light dosimetry software tool, which was implemented to individualize the light dose and compensate for any treatment. The spatially resolved spectroscopy was utilized to assess the effective attenuation coefficient of the target tissue or organ. These data can be input to the AI calculation system, like the cimmino optimization algorithm, to calculate the light irradiation times, dosage, and intensity for the infection area (67). Wang et al. also studied the irradiance uniformity optimization for a photodynamic therapy treatment device with a 3D scanner, which compared the general and optimized light source. The optimized light source increased the effective irradiance area for optimizing the light dosimetry to realize intelligent and personalized treatment (68).

b. Tissue oxygenation is another dosimetric factor involved in the PDT process (69). It is important to have the measurement of oxygenation levels for the surrounding treatment area, before and after the PDT treatment. McIlroy et al. identified that the spatial measurement of oxygen levels during PDT using time-resolved optical spectroscopy (ML algorithms). The phosphorescent compound, palladium meso-tetra(carboxyphenyl)porphine, was used in this study for the measurement of *in vivo* microvascular oxygen tensions in a rat liver during the PDT process. Time-resolved phosphorescence detection and oxygen tensions were determined by the phosphorescence lifetimes using Stern-Volmer analysis. This technique contributed to improving the dosimetry of PDT (70). Monitoring tumor oxygenation is vital, as hypoxic microenvironments often limit PDT efficacy and contribute to treatment resistance in oncology. Szczygieł et al. indicated that electron paramagnetic resonance (EPR) spectroscopy (ML algorithms) enabled the study of the quantitative and sequential measurements of partial pressure of oxygen (pO_2_) in an animal with PS in tumor tissue during the PDT process. It monitored the PDT-induced changes in tumor oxygenation and was used to identify potentially resistant tumors (71).

c. Inherent properties of PS contain absorbing specific light wavelengths and emission, converting light energy to ROS with singlet oxygen (Type II) or free radicals (Type I) for the therapeutic effects during the PDT process. This should result in minimal dark toxicity with high quantum yield for the generation of ROS, and accumulates in target tissues (72). Therefore, PS is an indispensable component of PDT because it determines the selectivity, efficiency, and safety of the treatment (73). The ideal characteristics of PS include: it has (i) strong absorption in specific wavelength ranges, often in the visible or near-infrared (NIR) region (600 to 800 nm) for the tissue penetration (74); (ii) high quantum yield for the generation of ROS and maximizes cytotoxicity (75); (iii) select localization for determining the distribution of PS in targeting tissue (76); (iv) minimal dark toxicity to ensure the distribution of PS is non-toxic and safety during the PDT process (77); (v) stable chemical property, and easy to dissolve under physiological conditions for intravenous or topical use (78). Thus, AI serves as a powerful tool to enable rational design, helping to optimize critical factors and fulfill the requirements for effective PDT agents by predicting photophysical properties, modeling biocompatibility, and screening large chemical libraries to prioritize promising candidates. AI establishes a model for absorption spectra, predicts ROS yields, simulates tissue distribution, and evaluates toxicity profiles by leveraging ML and DL algorithms. This computational approach accelerates the discovery of novel PS candidates for enhancing clinical translation and efficacy, to ensure that PDT achieves its precision, accuracy, and safety in diverse therapeutic effects. The rational PS design ensures tumor selectivity, minimizes systemic toxicity, and enhances therapeutic outcomes for cancer applications.

Chen et al. reported a unified active learning framework for PS design. In this study, the framework integrated three principal components, such as (i) a hybrid quantum mechanics–machine learning pipeline; its function to generate chemically diverse molecular datasets and preserve quantum-level accuracy at substantially reduced computational cost; (ii) a graph neural network architecture with uncertainty quantification, to enable robust predictions and reliable model performance; (iii) novel acquisition strategies, which was the dynamically balance broad exploration of chemical space with targeted optimization of key photophysical properties. These elements establish the AI data-driven platform for the accelerated discovery of novel PS from optoelectronic materials, with immediate relevance to solar energy conversion and wide applicability across emerging photonic technologies (79).

Xu et al. (80) developed a self-improving discovery system that integrates first-principles simulations with Bayesian optimization-based deep learning, improving prediction accuracy for singlet–triplet splitting and enabling efficient identification of high-performance PSs. Wang et al. (81) introduced the AI-accelerated PS innovation (AAPSI) framework, a closed-loop workflow combining expert knowledge, scaffold-based molecular generation, and Bayesian optimization. This iterative system ensured structural novelty, synthetic feasibility, and accelerated the discovery of novel PSs. These AI-driven frameworks are directly applicable to oncology, enabling the rapid identification of photosensitizers with optimal tumor penetration, ROS yield, and biocompatibility. Together, these advances highlight diverse AI-driven strategies for rational photosensitizer design. These are recent advances that highlight diverse AI-driven strategies for the designation of PS.

### 9.2 Clinical outcomes for AI-PDD/PDT against cancer

PDD integrates with AI, strengthening cancer detection and treatment planning. Preliminary studies suggest improved tumor visualization and intraoperative guidance, but clinical validation remains limited. For example, IR employs organic light-emitting diodes (OLEDs) with NIR to increase the tumor-targeting effectiveness, and exceeds the results of standalone treatments (82). PDD and PDT are the diagnostic–therapeutic continuum, PDD has a unique advantage in the early stage for sensitive detection, which PDT cannot achieve. However, they enhance their effectiveness with the help of AI support that is much better than being alone, because AI enhances the effectiveness of PDD and PDT by applying ML and DL models for data collection and analysis. PDD has been widely applied to penile carcinoma, prostate cancer, kidney tumors, and urethral condylomata, focusing on diagnosis, detection, and imaging (83). The applications highlight AI-PDD’s versatility across urological cancers, where early detection and precise imaging show promise for reducing recurrence risks. However, survival outcomes have not yet been conclusively demonstrated. Meanwhile, this synergy transforms PDD and PDT from isolated modalities into a closed-loop system, complementary to each other, that promotes the diagnosis and treatment of any disease.

PDT is the therapeutic intervention treatment, while therapeutic describes the beneficial effects of the intervention. The mechanism of PDD requires a light source and a PS for activation to produce fluorescence image detection and analysis, but PDT requires a light source and a PS for activation to generate ROS for tumor destruction. The role of AI in PDD is for the analysis of images, fluorescence interpretation, and lesion segmentation, while AI in PDT optimizes light dose, intensity, PS distribution, and treatment planning. For clinical outcomes, AI-PDD may provide guidance and margin delineation for early cancer detection, whereas AI-PDT may have personalized treatment to improve the effect of therapy on the tumor. Thus, AI-PDD supports diagnostic accuracy, while AI-PDT enhances therapeutic precision, together forming a closed-loop oncology system. AI-PDD must have a high-quality annotated dataset to help with the detection for image analysis, while AI-PDT must have biophysical modeling and depend on the light source and PS for therapy.

### 9.3 Current AI-PDD/PDT research

Small sample sizes in AI-PDD/PDT datasets lead to poor reproducibility, like lack of study resources, ethical considerations in animal studies, and without diagnosis. By employing emergent self-organizing maps (ESOMs), the algorithm adapts to complex, high-dimensional data structures and generates representative outputs for each original data point. This process effectively augments sample sizes in small or rare case datasets, even under conditions of limited training data. This approach directly addresses the challenges posed by small sample sizes in biomedical research, providing a robust tool to enhance the reliability and reproducibility of scientific findings (84). This is particularly for rare tumor subtypes, where limited datasets hinder robust AI model training in cancer research. Kolla reported that there was a retrospective study, and recommended for breast, prostate, and lung cancer treatment generated by OpenAI’s commercial LLM, ChatGPT, aligned with the standard-of-care set by the National Comprehensive Cancer Network (NCCN) to encode clinical knowledge, automate medical documentation (e.g., informed consent), enhance telemedicine interactions, and assist in clinical trial enrollment (85–87). These examples demonstrate how AI can streamline oncology workflows, from documentation to patient stratification, aligning with evidence-based cancer guidelines. Besides small sample sizes, the lack of clinical validation for AI-PDD/PDT is another issue. The validation in larger studies may inform the development of safety frameworks and quality indicators for maintaining high standards of care (88). Larger validation studies are required to establish safety frameworks, quality indicators, and regulatory acceptance of AI-PDD/PDT for oncology.

### 9.4 Translational challenge for AI-PDD/PDT

AI models have evolved rapidly, which may affect the long-term relevance, as architectures and larger datasets can quickly surpass current benchmarks in PDD/PDT. Present investigations are based on ML and DL frameworks such as CNNs, RF, and PBPK/PD modeling (14, 26, 33). However, emerging approaches including transformer networks and multimodal learning have already demonstrated superior performance in biomedical imaging and predictive modeling (34). These findings are summarized and interpreted as provisional, emphasizing the requirements for continuous benchmarking, multicenter validation, and adaptive clinical protocols to sustain clinical utility (8, 9).

The integration of AI into healthcare or clinical adoption holds great promise for enhancing patient outcomes through accelerated diagnostics and individualized treatment strategies. Nevertheless, the limited interpretability of many AI models poses substantial barriers to their clinical implementation, such as the black-box nature of deep learning models and the variability in performance across different clinical settings (89). This is a complex model with deep learning and large language to operate with opaque internal mechanisms. Clinicians and regulators struggle to trust outputs without clear reasoning. Model interpretability is regarded as a critical requirement, particularly in high-risk domains such as healthcare, where reliability is paramount (90). The interpretability is especially critical, as treatment decisions directly affect survival outcomes and long-term patient quality of life in oncology. AI systems may yield inconsistent or contradictory outputs in clinical practice, which can result in severe consequences across diverse cultural and situational contexts. Employing interpretable methodologies enables transparency in decision-making, facilitates the tracking of individual predictions, and provides mechanisms to regulate model behavior. Thus, it should use the ML/DL models correctly to make a final decision. The typical interpretability models include ante-hoc (intrinsic), post-hoc, global, and local approaches in clinical adoption (**Table 6**), which refer to interpretability addressing the model behavior, whereas local is the case-specific explanation of individual predictions. The local interpretability ensures clinicians can trust AI-PDD/PDT outputs when guiding surgical excision or tailoring PDT dosimetry for cancer care. It is necessary to select the most appropriate model to ensure that clinical datasets are explained accurately and reliably.

**Table 6:** Types of interpretability models in clinical adoption.

Type of interpretability model	Description	Comments	Reference
Ante-hoc (intrinsic)	Models are designed to be transparent at the beginning, for example, decision trees and linear regression	Easy to interpret, but may trade off accuracy	(91, 92)
Post-hoc	Explanations generated after training (e.g., SHAP, LIME)	High-accuracy models can be explained, but these may not fully match internal logic	(93)
Global	Explain the overall model behavior across the dataset	Useful for understanding general feature importance	(92)
Local	Discuss the specific decisions or predictions for individual cases	Narrower scope, feasible, inexpensive, highly relevant in clinical settings	(93)

Current computational models provide insights into the TME during PDT, but the ability to capture dynamic complexity remains limited. Spheroid-on-chip and microfluidic systems can mimic normoxic and hypoxic conditions, allowing better prediction of oxygen depletion and ROS generation (91). Physiologically based PBPK/PD simulations and ROS kinetic models approximate PS distribution, light penetration, and oxygen consumption, offering mechanistic understanding of therapeutic outcomes (26, 33). However, these models oversimplify tumor heterogeneity, failing to screen stromal interactions, vascular remodeling, and immune modulation, which are critical determinants of PDT efficacy (92). Temporal changes in oxygenation and metabolic adaptation are difficult to reproduce in silico, limiting translational accuracy. This should be interpreted as provisional tools to guide hypothesis generation rather than definitive predictors. Integration of immune, stromal, and vascular dynamics is required, coupled with multicenter validation to enhance clinical relevance (93).

There are several translational challenges for AI-PDD/PDT. These must be addressed before routine clinical adoption. The challenges include interpretability, validation, regulation, and workflow integration.

Explainable AI (XAI) and interpretability for DL models such as CNNs and YOLO remain “black boxes,” limiting clinician trust. The frameworks have gradient-weighted class activation mapping, Shapley additive explanations, and local interpretable model-agnostic explanations that provide visual or statistical explanations of model decisions and image regions or quantify feature importance. Recent reviews emphasize the XAI for regulatory approval and clinical trust, but current applications remain largely experimental (105, 106).

External validation and multicenter prospective studies for most AI-PDD/PDT evidence are retrospective or preclinical. Large multicenter prospective trials are required to confirm reproducibility and generalizability across diverse patient populations. For example, a multicenter validation study of an AI-empowered blood-based test for multi-cancer early detection demonstrated external benchmarking for clinical credibility (107).

Regulatory approval pathways for AI in healthcare are evolving. The FDA issued draft guidance in 2025 for AI/ML in drug and biologic development, emphasizing Good Machine Learning Practice and risk-based validation (108). The EMA’s Reflection Paper (109) and the EU AI Act (Regulation 2024/1689) (110) provide European standards for transparency and oversight. These frameworks provide for continuous monitoring and post-market surveillance of AI-enabled medical devices. Ethical considerations include equity and transparency, while algorithm bias and data privacy remain critical barriers to safe deployment (Section 9.5 for detailed discussion).

Integration into clinical workflows with AI systems must integrate seamlessly into oncology guidelines. Challenges include graphics processing unit infrastructure, clinician or healthcare workers training, and interoperability with hospital systems. There were more than 120 FDA-cleared AI tools embedded in 2025 oncology, but generalizability and workflow disruption remain barriers (111). Successful integration requires interdisciplinary collaboration and adaptive clinical protocols.

### 9.5 Ethical considerations

The use of AI in healthcare may cause ethical dilemmas, such as privacy and data protection, informed consent, and human connection in medical consultations (94). These dilemmas are amplified because cancer patients often undergo repeated imaging, biopsies, and AI-assisted interventions, raising heightened concerns about consent and data security in oncology. Privacy and data protection, as social media is part of the AI aspects, and ensuring the safety of the patients’ data is still a significant concern when using robots. Patients have the right to be informed of their diagnoses, health status, treatment process, therapeutic success, test results, costs, health insurance share, or other medical information, and all of these are involved in the AI healthcare applications and raise the problem concerned according to the autonomy principle (95). Respecting autonomy is critical, as patients must weigh complex treatment options, potential side effects, and long-term survival outcomes when consenting to AI-assisted PDT for cancer care. Healthcare providers may seek consultation from or provide consultation to their colleagues, which is not possible in autonomous (robotic) systems. It seems unlikely that patients accept “machine-human” medical relations instead of “human-human.” This tension is particularly sensitive in oncology, where trust and empathy are central to patient–clinician relationships during emotionally challenging cancer treatment journeys. Although there was evidence that robot-assisted, image-guided PDT minimizes risks compared to open surgery by enabling precise, targeted light delivery to tumor sites while sparing surrounding tissue (96), which can offer more precise treatment for patients (97). This precision is vital for minimizing collateral damage in delicate tumor sites, but it also raises ethical questions about accountability when robotic systems are involved in oncology. Nonetheless, several challenges remain, including integration of robotic systems within the operating room environment, limitations in robotic perception, and deviations in needle insertion. In conclusion, image-guided, robot-assisted percutaneous puncture raises important ethical and safety considerations for patients (98). Thus, ethical frameworks must balance innovation with patient rights, ensuring that AI-PDD/PDT adoption enhances care without compromising trust, autonomy, or safety in cancer applications.

Meanwhile, AI PDD/PDT demonstrates strong potential to enhance diagnostic precision and therapeutic planning. However, most of the current evidence originates from computational modeling, retrospective analyses, animal studies, or proof-of-concept investigations. Clinically validated applications remain limited, with only a few multicenter trials reporting improved detection rates. The claims of improved patient outcomes should be interpreted cautiously. Future research should focus on prospective, multicenter validation and standardized AI reporting to bridge the translational gap between preclinical innovation and routine clinical practice.

## 10 Conclusions

AI has rapidly grown in recent years, which offers significant potential to enhance PDD and PDT by the integration of ML and DL frameworks. AI enables computational predictions that refine PDT variables, streamline treatment workflows, and accelerate the rational discovery of high-performance PSs. These address limitations of conventional PDT, such as empirical dosimetry, variability in tissue oxygenation, and restricted monitoring of ROS.

However, the current evidence base remains heterogeneous. Most existing evidence consists of conceptual proposals or preclinical proof-of-concept studies. Only a limited number have progressed to retrospective analyses or early-stage clinical validation. Most AI-PDD/PDT studies remain at the conceptual or preclinical stage, with only limited retrospective analyses in bladder and oral cancers showing promising translational potential for cancer applications. The translational maturity of AI-assisted PDD/PDT should be interpreted cautiously. Promising advances, such as adaptive light activation, predictive modeling of oxygen and ROS dynamics, and AI-driven robotic infusion systems, are still largely confined to experimental or pilot settings. Adaptive light activation and predictive oxygen/ROS modeling are especially critical for overcoming tumor heterogeneity and hypoxia, two major barriers to PDT efficacy in the oncology. The rational design of PS is another area where AI shows promise, particularly in modeling absorption spectra, predicting ROS yields, simulating bio-distribution, and evaluating toxicity profiles. Most of these approaches remain at the discovery or optimization stage rather than clinical deployment. The rational PS design guided by AI accelerates the discovery of tumor-selective agents with improved penetration, reduced toxicity, and enhanced ROS yield for cancer therapy.

In summary, AI-assisted PDD/PDT represents a diagnostic–therapeutic continuum, but the field is still in an early translational phase. Prioritizing systematic validation, standardized protocols, and rigorous clinical trials are the future works to move beyond descriptive promise toward reliable, evidence-based integration into clinical practice. Meanwhile, this should also explore systematic integration of Chinese medicine diagnostic modalities with AI-PDD/PDT to ensure the Western and Chinese perspectives are represented in translational research. Ultimately, AI-PDD/PDT against cancer represents a diagnostic–therapeutic continuum with immense promise, but requires systematic validation, multicenter trials, and integration of Western medicine diagnostic frameworks to achieve reliable clinical adoption.

Generally, AI-assisted PDD/PDT remains at an early translational stage. Future research must prioritize systematic validation through multicenter trials, standardized dosimetry protocols, and harmonized imaging workflows to ensure reproducibility across diverse patient populations. Robust benchmarking against conventional method is implied to avoid overstating clinical maturity. The explainability and regulatory compliance are critical for clinical adoption, as opaque algorithms may hinder physician trust and patient safety. Integration of multimodal datasets should expand predictive accuracy but requires careful evaluation of interoperability and ethical considerations. Addressing tumor heterogeneity, hypoxia, and variability in PS uptake demand adaptive AI models that generalize across experimental settings. Ultimately, AI-PDD/PDT should be viewed as a promising but evolving paradigm, where realistic progress depends on rigorous evidence, transparent validation, and collaborative translational research rather than premature claims of widespread implementation.

## 11 Future aspects

AI supports the design of PS in PDD and PDT that become nanosized with the help of nanotechnology with ML, and clustered regularly interspaced short palindromic repeats in combination with PDD/PDT in cancer detection, research, and therapy (99). Nanosized PSs combined with AI-PDD/PDT offer enhanced tumor selectivity, deeper tissue penetration, and reduced systemic toxicity, making them highly promising for precision cancer therapy in oncology.

Regarding AI-PDD, Elbassiouny et al. reported the PDD of parathyroid glands with nano-stealth aminolevulinic acid liposomes. This study compared nano-stealth liposomes and nano-liposomes with free ALA solution in rats. Liposomal formulations enhanced intraoperative identification of parathyroids by increasing fluorescence contrast, thereby reducing postoperative side effects (100). Although focused on parathyroid visualization, similar nano-liposomal strategies are being adapted for oncology to improve intraoperative tumor identification and minimize recurrence. Lin et al. synthesized the PLZ4-nanoporphyrin (PNP) and integrated it with PDD, which has the efficiently fluorescence signal of PNPs and selectively increased in bladder cancer cells but not normal urothelial cells *in vitro* and in an orthotopic patient-derived bladder cancer xenograft (PDX) model (101). This demonstrates how AI-PDD can selectively enhance tumor fluorescence in bladder cancer, supporting translational progress toward clinical adoption.

For AI-PDT, Kanelli et al. reported a machine learning-optimized system for pulsatile, photo, and chemotherapeutic treatment using near-infrared responsive MoS_2_-based microparticles in a breast cancer model. This studied a therapeutically relevant for patients in need of recurring cycles of treatment on small tumors through ML. It provided precise localization and low invasiveness, as well as being non-cross-resistant with other treatments (102). This approach highlights how AI-optimized PDT can complement existing therapies, offering new options for resistant or recurrent tumors in oncology. The next milestone may be the interface between AI and PDT for its clinical applications, such as developing the interface model between patient and clinician. It may involve data integration and the clinician’s decision. However, AI may miss the data, and imputation strategies influence the predictive performance for PDT treatment (103). Developing robust clinician and AI interfaces will be critical to ensure accurate data integration, minimize imputation bias, and support safe treatment decisions for cancer care. Meanwhile, the data extraction in AI was done with a large language model. Some classification errors required subjective judgment by humans (104). These ethical issues need to be considered, which are magnified, as AI-PDD/PDT directly influences life-saving decisions, requiring transparent algorithms, patient trust, and regulatory oversight in the future oncology.

Before AI-PDT can be incorporated into standard oncology guidelines, several levels of evidence are required. Robust multicenter randomized controlled trials must demonstrate reproducible improvements in patient outcomes, including survival, recurrence rates, and quality of life (8, 9). Standardized protocols for PS dosing, light delivery, and AI-driven dosimetry optimization to ensure consistency across institutions (14, 26). Regulatory validation is transparent reporting of algorithm performance, interpretability, and safety in diverse clinical settings (89, 90). Comparative effectiveness studies should benchmark AI-PDT against existing standards of care, such as chemotherapy or radiotherapy, to establish cost-effectiveness and clinical superiority (33, 34). The integration of real-world evidence from registries and longitudinal cohorts is critical to confirm generalizability. This layered evidence base can support AI-PDT progress from promising innovation to guideline-endorsed practice in oncology.
