# Supplementary material for: Artificial intelligence-assisted photodynamic diagnosis and photodynamic therapy against cancer
Source: Front Oncol. 2026 Aug 25;16:1932816. doi: 10.3389/fonc.2026.1932816 (PMC13562838; doi:10.3389/fonc.2026.1932816)
Supplement: Supplementary file 1 [file Table1.docx]

**Narrative Review Checklist**

| **Section/Topic** | **Item No** | **Item** | **Reported on Section/Paragraph** |
| --- | --- | --- | --- |
| **TITLE** | | | |
| Title | 1 | Identify the report as a Narrative Review or Literature Review. | Title: Artificial Intelligence-Assisted Photodynamic Diagnosis and Photodynamic Therapy against Cancer |
| **ABSTRACT** | | | |
| Structured summary | 2 | Provide a structured summary with the subsections as: objective, background, methods, conclusion. | Abstract – Background, Methodology, Results, and Conclusions |
| **INTRODUCTION** | | | |
| Rationale/background | 3 | Describe the rationale for the review in the context of what is already known. | Introduction – AI in medicine, ML/DL, importance of PDD/PDT |
| Objectives | 4 | Specify the key question(s) identified for the review topic. | Introduction – This review article discusses the different areas of AI-assisted PDD/PDT, including operation, advantages, recent research progress with examples, and its future aspects |
| **METHODS** | | | |
| Research selection | 5 | Specify the process for identifying the literature search (eg, years considered, language, publication status, study design, and databases of coverage). | Abstract – Methodology: databases (PubMed, Scopus, Web of Science, CNKI, etc.), 2003 to 2025, no language restriction with keywords, inclusion/exclusion criteria |
| **DISCUSSION/SUMMARY** | | | |
| Narrative | 6 | Discuss: 1) research reviewed including fundamental or key findings,  2) limitations and/or quality of research reviewed, and 3) need for future research. | Overview, AI-assisted PDD, AI-assisted PDT, Advantages, Research process – includes findings, limitations (talent shortages, inspection standards, resource wastage), and future research (translational opportunities, personalized treatment) |
| Summary | 7 | Provide an overall interpretation of the narrative review in the context of clinical practice for health professionals, policy development and implementation, or future research. | Conclusions and future aspects – AI-PDD/PDT improves accuracy, safety, and efficiency; future milestone for the development of clinical interface |

Table S1. 56 selected studies were included in this review.

| **Ref. No.** | **First Author** | **Year** | **Study Focus** | **AI Method** | **Type of Cancer / Application** |
| --- | --- | --- | --- | --- | --- |
| 1 | Bellini V | 2022 | AI principles for clinicians | General AI | Clinical practice |
| 2 | Prathaban K | 2025 | AI in imaging & drug response | ML/DL | Oncology |
| 3 | Samala RK | 2024 | AI in medical imaging | ML/DL | Translational imaging |
| 4 | Toma A | 2022 | Deep learning in medicine | DL | General medicine |
| 5 | Rana M | 2022 | ML/DL for medical image analysis | ML/DL | Diagnosis |
| 6 | Aljohani M | 2025 | Hierarchical ML for kidney tumors | ML | Oncology |
| 7 | Bekbolatova M | 2024 | AI in healthcare & ethics | General AI | Healthcare |
| 8 | Blichert-Refsgaard L | 2025 | PDD at TURB in NMIBC | PDD | Bladder cancer |
| 9 | Zumbraegel A | 2003 | Early bladder carcinoma detection | PDD | Bladder cancer |
| 10 | Olszowy M | 2023 | PDT/PDD strategies & nanotech | PDT/PDD | Cancer |
| 11 | Silva CR | 2025 | PDT and radiotherapy sequencing | PDT | Breast cancer |
| 12 | Chen Z | 2025 | Lysosome-targeted PDT | PDT | Colorectal cancer |
| 14 | D’Adderio L | 2025 | AI transforming diagnosis | ML/DL | Oncology |
| 15 | Mofokeng M | 2025 | AI-driven PDT | ML/DL | Cancer |
| 16 | Wan W | 2025 | PDD and autofluorescence imaging | PDD | Tumor diagnosis |
| 17 | Kong Z | 2025 | AI-assisted diagnostic system | DL | Oncology |
| 18 | Del Valle AC | 2025 | AI in digital pathology | ML/DL | Cancer diagnosis |
| 19 | Paul A | 2026 | AI in PDT | ML/DL | Cancer |
| 20 | Kubrak T | 2022 | PDT in bladder cancer | PDT | Bladder cancer |
| 24 | Fekrazad S | 2025 | AI in photomedicine | ML/DL | PDT |
| 25 | Algorri JF | 2021 | Light technology in PDT | PDT | Cancer |
| 26 | Wu K | 2024 | AI-PBPK modeling | ML | PDT dosimetry |
| 32 | Garabaghi FH | 2023 | Oxygen dynamics modeling | ML | PDT efficacy |
| 33 | Ji Z | 2025 | ROS prediction | ML | PDT cytotoxicity |
| 36 | Nkune NW | 2024 | AI integration with PDT/PDD | ML/DL | Oncology |
| 37 | Saeidi T | 2025 | PS heterogeneity in PDT planning | ML | Colorectal cancer |
| 38 | Arora P | 2024 | AI in drug discovery | ML | PS benchmarking |
| 39 | Sarbadhikary P | 2021 | Theranostic PS agents | ML/DL | Imaging-guided PDT |
| 40 | Somani BK | 2010 | PDD for UTTCC | PDD | Bladder cancer |
| 41 | Kim H | 2025 | AI-guided laparoscopy | DL | Peritoneal cancer |
| 42 | Yamashita T | 2022 | AI-based endoscopic PDD | DL | Gastric tumors |
| 43 | Yamamoto S | 2023 | DL to reduce false positives | DL | Bladder cancer |
| 44 | Rivera-Piza A | 2025 | AI-guided laparoscopy | DL | Peritoneal cancer |
| 45 | Pourhajibagher M | 2025 | AI in antimicrobial PDT | ML | Antimicrobial therapy |
| 46 | Kim WS | 2022 | AI-enabled PDT telemetry | DL | Colorectal cancer |
| 47 | Chen W | 2025 | AI-driven PS discovery | ML | RNA-targeted PDT |
| 48 | Rahman MA | 2024 | DL insights into PDT effects | DL | Cancer cells |
| 49 | Diao B | 2025 | ML-assisted PS design | ML | Theranostic PDT |
| 50 | Lv S | 2023 | DL evaluation of PDT efficacy | DL | Tumor response |
| 51 | Yoo TK | 2022 | DeepPDTNet outcome prediction | DL | Ophthalmology |
| 52 | Stenzl A | 2011 | Clinical trial of fluorescence cystoscopy | PDD | Bladder cancer |
| 53 | Ding Q | 2026 | Intelligent photosensitizers | ML/DL | PDT |
| 54 | Ali N | 2021 | DL classification in cystoscopy | DL | Bladder cancer |
| 56 | Mutaguchi J | 2022 | AI segmentation of cystoscopic images | DL (UNet) | Bladder cancer |
| 58 | Ikeda A | 2020 | AI support for cystoscopic diagnosis | ML | Bladder cancer |
| 59 | Lee JY | 2015 | Comparative imaging technologies | ML | NMIBC |
| 60 | Tejedor-Estrada R | 2012 | ANN for PS localization | ANN | Solid tumors |
| 61 | Yassine AA | 2021 | ML for optical property recovery | ML | PDT dosimetry |
| 62 | He MJ | 2023 | AI-assisted DNA cytology | ML | Oral leukoplakia |
| 95 | Quon H | 2011 | PDT in head & neck dysplasia | PDT | Oncology |
| 96 | Huang X | 2024 | PDT and robotic surgery | ML integration | Colorectal cancer |
| 98 | Chota A | 2025 | Nano-enhanced PDT and ML | ML | Breast/colorectal cancers |
| 99 | Elbassiouny S | 2018 | Nanoliposome-based PDD | ML | Parathyroid glands |
| 100 | Lin TY | 2016 | Theranostic nanoporphyrins | ML | Bladder cancer |
| 101 | Kanelli M | 2024 | ML-optimized multimodal therapy | ML | Breast cancer |
| 104 | El-Sadek MZ | 2025 | Emerging PDT innovations | ML/DL | Cancer treatment |
